# Two-year Results of an Emergency Department Night Shift Buy-out Program

**DOI:** 10.5811/westjem.20303

**Published:** 2024-12-31

**Authors:** Charlotte W. Croteau, Joshua N. Goldstein, Lauren Nentwich, Ali S. Raja, Michael VanRooyen, Joshua J. Baugh

**Affiliations:** Harvard Medical School, Massachusetts General Hospital, Department of Emergency Medicine, Boston, Massachusetts

## Abstract

**Introduction:**

Emergency physicians have the highest rates of burnout among our physician peers, with prior literature suggesting clinician schedules can play a significant role in burnout. We assessed our transition from a tenure- and age-based paradigm to an egalitarian, night shift buy-out program that allows schedule flexibility for physicians at all stages of their careers.

**Methods:**

The night shift buy-out program was implemented in the emergency department (ED) of an academic, quaternary-care center that treats approximately 100,000 adult patients annually with 56 faculty emergency physicians. We sought to create a cost-neutral program, carefully balancing incentives between nocturnists and those wanting to reduce allotted night shifts. Ultimately, the program was designed to allow all faculty to buy out of any number of nights for $500 per night shift, with the funds generated used to increase nocturnist salaries. We analyzed two years of the program (July 2022–June 2024) to assess trends in night shift buy-outs, the primary outcome. We also conducted an all-faculty survey after the program’s first year to gauge sentiments about the program.

**Results:**

Over two years, 22 faculty (42%) fully bought out of nights; an additional 10 (15%) bought out of some nights. By year two, the program could grant all faculty their preferred night-shift allotment. Faculty who bought out fully had worked longer in EM on average, worked fewer clinical hours per year, were more likely to be associate/full professors, and were less likely to be women. Nocturnists had the highest mean clinical hours of the four groups, had the lowest average tenure, and were least likely to be associate/full professors. A total of 86% of faculty responded to the survey, to which more than 80% of those buying out reported that reducing the night-shift burden was either “very important” or “critical for continuing in this job.”

**Conclusion:**

Our academic ED transitioned from a tenure- and age-based, overnight shift paradigm to an egalitarian buy-out program that allows physicians flexibility at all career stages. This approach could improve career satisfaction and reduce burnout among emergency physicians.

Population Health Research Capsule What do we already know about this issue?

*Emergency physicians (EP) have the highest rates of burnout among physicians, with prior literature suggesting clinician schedules can play a significant role.*

What was the research question?

*Does a night shift buy-out program improve physician career satisfaction and reduce burnout among emergency physicians?*

What was the major finding of the study?

*More than 80% of physicians buying out of night shifts reported this was either “very important” or “critical for continuing in this job.”*

How does this improve population health?

*We transitioned from a tenure -and age-based overnight paradigm to an egalitarian buy-out program. This could improve career satisfaction and reduce burnout among EPs.*

## INTRODUCTION

Emergency physicians (EP) have the highest rates of burnout among our physician peers.^1^ Maslach defines burnout as the triad of depersonalization, emotional exhaustion, and decreased sense of personal accomplishment.^2^ While much effort has been directed toward finding meaningful solutions to counteract burnout in our field, the problem only appears to be worsening. In the 2022 Medscape Physician Burnout and Depression Report, there was a significant increase in burnout among EPs between 2021 and 2022, from 43% to 60%.^3^

Prior studies suggest that one factor affecting burnout in medicine is a physician’s clinical schedule. Night shifts have been shown to negatively influence job satisfaction among EPs.^4^ While “exhaustion” often refers to “emotional exhaustion” in the burnout literature, sleep deprivation has been identified as a contributing factor to decreased personal well-being, lower quality of care, and harmful health outcomes.^5^ ^–^ ^7^ Sequential night shifts, in particular, have been associated with reduced cognitive performance in EPs, and shift work in general has been associated with a disruption in circadian rhythms.^8^ While providing care around the clock is fundamental to the duty of emergency departments (ED), the 24/7 shifts also likely contribute to the high burnout rates among EPs.

Despite concerns about night shifts, some physicians elect to work only overnight. Reasons cited for choosing a nocturnist schedule include more independence, more time with family, higher salary, and scheduling flexibility.^9^ Hiring dedicated nocturnists may allow some physicians to avoid undesirable night shifts while enabling others to opt into a primarily night-shift schedule. According to Maslach’s theory of burnout, increasing employees’ control over their work can decrease burnout; allowing physicians to opt in or out of nights may be a win-win for everyone’s well-being.^2^ In our academic ED, we transitioned from a tenure- and age-based nights paradigm to an egalitarian, night shift buy-out program that allows physicians flexibility at all career stages. Goals included budget neutrality, improving equity (by giving all faculty, irrespective of age, equal options), and increasing agency (by giving all faculty opportunity to adjust their schedule to match their own needs). This study assessed patterns in night shift buy-outs and EPs’ sentiments about the program.

## METHODS

### Study Design and Setting

This retrospective, cohort, institutional review board-exempt study was conducted in an ED within an academic, quaternary-care center that sees approximately 100,000 adult patients annually. Our department includes both a pediatric section and an adult section; this study focuses on the staffing of the adult section. The adult attending group in the study ED comprises 56 faculty and 7–9 fellows per year. Only faculty were eligible for the overnight buy-out program; fellows did not have this option. There are 13 adult attending shifts per 24-hour period, three of which are overnight shifts. Historically in our department, attending night shifts were allocated based on academic rank, with an additional option to stop working nights altogether at age 60, regardless of academic status.

### Night Shift Buy-out Program

We sought to create a new program where all faculty could buy out of night shifts. To facilitate this, we recognized the need to hire more nocturnists. Our goal was a cost-neutral program, requiring careful balancing of incentives. Therefore, several rules were established for the program.

All faculty members were eligible after one year of service, regardless of academic rank; over two years our program evolved into the system described below. To determine how many baseline night shifts EPs in our department owed, we used a prorated equation based on total clinical time, adjusted each year depending on the makeup of the attending roster and scheduling needs of the department. Most physicians in our department owed between 12–24 night shifts per year. Physicians were then offered the option of reducing their number of night shifts for the year in exchange for a salary reduction of a specific dollar amount ($500) per night shift. This number was chosen because it reflects the pay differential per shift for a nocturnist in our group. Physicians could buy out of any number of night shifts; they could decrease their nights by a single shift, buy out of all night shifts, or anything in between. Total annual clinical hours owed by these physicians did not change: bought-out night shifts were instead converted to days and evenings. The funds generated by bought-out nights were used to increase the salaries of nocturnists compared to non-nocturnist attendings, keeping the program cost-neutral. Nocturnists could also pick the exact days they wished to work, providing total schedule control as an added incentive.

We did maintain an additional option for faculty over the age of 60. These faculty were given a choice regarding how they would like to decrease their nights: they could buy out as above, work more weekends instead of nights in a 1:1 proportion, or increase their total clinical hours in exchange for decreasing nights.

The EM night shift buy-out program was originally implemented on July 1, 2022. All faculty had to commit to their buy-out plan for one full year, with an option to modify their choices at the end of each year. While we are currently in year two at the time of writing, all buy-out decisions have already been made for the program’s full second year.

### Outcome Measures and Data Collection

We analyzed two years of program data to assess patterns in night buy-outs among the faculty, the primary outcome measure. We also evaluated the demographic characteristics of participating EPs separated into four groups: those who 1) had full buy-out from nights; 2) had partial buyout of nights; 3) had no buy-out of nights; and 4) were nocturnists. Demographics assessed included the following: years in EM, defined as years since medical school graduation; academic rank, stratified as clinical instructor or assistant professor vs associate or full professor; clinical hours worked, expressed as a percentage of a full-time clinical requirement in our ED; and sex, defined as male, female, or other. This data was assembled from our faculty hiring database as well as our department’s scheduling software, with analyses performed in Excel (Microsoft Corporation, Redmond, WA).

We also performed a survey during the program’s first year that included all EM faculty. This annual electronic survey typically assesses the well-being of our department. In the winter of 2022, we added the following question with multiple-choice, Likert scale answer choices for faculty who bought out of at least some night shifts: “How important is it for you to be able to decrease your night shift burden?” Answer choices included the following: not at all important; slightly important; moderately important; very important; and critical for continuing in this job. Given the small sample size, only descriptive statistics were performed.

## RESULTS

### Trends in Night Buy-outs

By the end of the program’s first year, our department increased its nocturnist faculty roster from three to six attendings. In the first year, we could not allow attendings under the age of 60 to fully buy out of nights because of clinical coverage needs; those desiring full buy-out had their nights decreased by 75% rather than 100%. With three additional nocturnists hired, we could fully accommodate buy-out requests for year two. Over the two years, 22 faculty (42%) fully bought out of nights, while an additional 10 (15%) bought out of some night shifts. Seven of the 10 who chose a partial buy-out decreased their nights by 50%, while the other three bought out for fewer than 50% of their night shifts.

### Demographics of Physicians Buying Out

Faculty who bought out fully had worked in EM for slightly longer on average, had lower total required clinical hours per year, were more likely to be associate or full professors, and were less likely to be women (See Table for total faculty group characteristics, as well as demographics by buy-out category). Nocturnists had the highest mean clinical hours of the four groups and the lowest average tenure and were least likely to be associate or full professors.

**Table.** Demographic characteristics of faculty in each buy-out category.

	All faculty (N=56)	Full buy-out (n=22)	Partial buy-out (n=10)	No buy-out (n=18)	Nocturnist (n=6)
Female	30%	18%	40%	39%	33%
Mean clinical FTE	0.56	0.48	0.55	0.61	0.65
Mean years in EM	15	17	14	15	9
Associate or full professor	45%	68%	20%	44%	0%

*FTE*, full-time equivalent; *EM*, emergency medicine.

### Faculty Feedback About the Program

Overall, 48 of 56 faculty (86%) responded to the survey at the end of year one. Of the 32 faculty who bought out at least some nights, 26 (81%) responded to the survey. More than 80% of those buying out reported that the ability to reduce the night shift burden was either “very important” or “critical for continuing in this job” (Figure).

**Figure.**
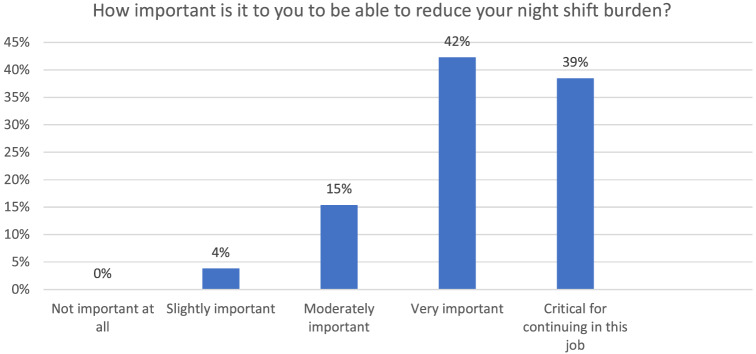
Responses from faculty (n=26) who used the buy-out program regarding the importance of buying out of night shifts as reported on our annual well-being survey.

## DISCUSSION

Night shifts contribute to burnout for some EPs and can detract from academic productivity.^4^ For others, working more night shifts can improve job satisfaction, mainly if doing so provides additional schedule control and compensation.^9^ Many departments currently reduce night shifts for physicians over a certain age, and the American College of Emergency Physicians has recommended accommodations for physicians in their pre-retirement years.^10^ More needs to be written about options for physicians to customize their overnight shifts at all stages of their careers. Our department created a program to allow any faculty member to buy out of night shifts, using the funds generated from buy-outs to incentivize nocturnist positions. Within two years of initiating the program, we allowed every faculty member with at least one year of service to buy out of their desired number of night shifts while hiring three additional nocturnists.

According to a national survey of academic EM leaders regarding policies around aging physicians, over half of the surveyed leaders reported decreasing or eliminating overnight shifts to accommodate aging physicians.^9^ While this is undoubtedly an important option for supporting longevity in EM, it does not address the impact of night shifts on younger physicians who, some studies suggest, experience the highest rates of burnout within medicine.^11^ During years when physicians may be raising young children while actively building their careers, increased schedule control might be beneficial for mitigating burnout.^12^ In addition, there may be non-age related reasons for some EPs to be disproportionately impacted by night shifts, including medical issues, mental health issues, or caregiver responsibilities, which can be hard to quantify.

A critical goal of the program was to promote equity across our faculty group. Programs that reduce night shifts based on age, academic rank or tenure may inadvertently propagate inequity, as physicians who are older, with longer tenure, or of higher rank may be more likely to be male and White than younger physicians.^13^ Despite offering the same buy-out option for all faculty, we did observe that those who bought out of nights entirely were more likely to be male, longer-tenured, and of higher academic rank than our group average. It should be noted that male faculty did have higher average academic rank than female faculty, but this did not fully explain the gender difference; among only high-ranking faculty, men were also more likely to buy out of nights than women. These trends may be related to historical preferences in our department or to financial realities for physicians at various career stages or with different family structures, among other potential explanations. Future research might explore reasons for differences in schedule preference among different demographic groups in EM.

For those participating in our program, most faculty reported that the ability to reduce nights was at least “very important,” with 39% indicating this was critical for continuing in their jobs. Prior research also suggests that simply giving people more control over their work can reduce burnout; there may be benefits of increased schedule choice for all faculty with a buy-out program, not just those who reduce night shifts.^2^ With record burnout in EM threatening career longevity for many, providing increased schedule control may be one strategy for improving faculty retention. Future research should examine whether self-reported answers to surveys like ours ultimately predict burnout and career decisions.

## LIMITATIONS

This study assessed our experience with a policy implemented for a single faculty group of physicians in one large ED. The trends discussed here may, therefore, be different from other departments. While this program could be adopted at other institutions, there may be unique considerations for implementation in other settings. We also observed the effects of the policy over a relatively short period and were only able to assess impact based on self-report; future work might examine longer intervals along with objective patterns of hiring and retention. We also did not specifically study the financial impacts of the program on our faculty. The personal impact of the loss of salary for attendings could be worth investigating in the future.

## CONCLUSION

In our large academic ED, we successfully transitioned from a tenure- and age-based night shift paradigm to an egalitarian night shift buy-out program that allows flexibility for physicians at all stages of their careers. Given the favorable results of the program, we have continued to allow this optional overnight buy-out for our faculty. This approach has the potential to improve career satisfaction, promote equity, and reduce burnout among emergency physicians.
